# Spatial Clustering of Rabies by Animal Species in New Jersey, United States, from 1989 to 2023

**DOI:** 10.3390/pathogens13090742

**Published:** 2024-08-30

**Authors:** Shamim Sarkar, Jaymie R. Meliker

**Affiliations:** Program in Public Health, Department of Family, Population, and Preventive Medicine, Stony Brook University, Stony Brook, NY 11794, USA; jaymie.meliker@stonybrookmedicine.edu

**Keywords:** rabies, spatial clustering, raccoons, foxes, groundhogs, hotspots, New Jersey

## Abstract

Identifying spatial clusters of rabies in animals aids policymakers in allocating resources for rabies prevention and control. This study aimed to investigate spatial patterns and hotspots of rabies in different animal species at the county level in New Jersey. Data on animal rabies cases from January 1989 to December 2023 were obtained from the New Jersey Department of Health and aggregated by county. Global Moran’s index (I) statistics were computed for each species to detect global spatial clustering (GeoDa version 1.22). Local Moran’s indicators of spatial association (LISA) were computed to identify local clusters of rabies. The results from the LISA analysis were mapped using ArcGIS Pro to pinpoint cluster locations. A total of 9637 rabies cases were analyzed among raccoons (n = 6308), skunks (n = 1225), bats (n = 1072), cats (n = 597), foxes (n = 225), and groundhogs (n = 210). A global Moran’s test indicated significant global spatial clustering in raccoons (*I* = 0.32, *p* = 0.012), foxes (*I* = 0.29, *p* = 0.011), and groundhogs (*I* = 0.37, *p* = 0.005). The LISA results revealed significant spatial clustering of rabies in raccoons and foxes in southeastern New Jersey and in groundhogs in northern New Jersey. These findings could guide the development of targeted oral rabies vaccination programs in high-risk New Jersey counties, reducing rabies exposure among domestic animals and humans.

## 1. Introduction

Rabies is a deadly viral disease that can infect all mammals, including domestic and wild animals. In the United States (U.S.) alone, nearly 100,000 animals are tested for rabies yearly [1], and over 4000 of these tests come back positive. The primary method of rabies transmission is through a bite or scratch from an infected animal [2], which can happen during encounters with wildlife such as raccoons or foxes and unvaccinated domestic animals. Rabies is rare in humans in the U.S., with an average of only 1 to 3 cases reported annually [3]. Animal bites are a much more common problem, with an estimated 4 million Americans bitten by animals each year [1]. Among these, approximately 60,000 individuals are estimated to have been exposed to rabies and need post-exposure treatment [1].

Rabies is not confined to specific regions; the virus is present throughout the U.S., posing a threat to humans and other animals. Wildlife are crucial in the spread of rabies in the U.S. [4,5]. Notably, New Jersey experiences a higher prevalence (95%) of the rabies virus in its wildlife [4]. Over 90% of reported rabies cases in New Jersey involve wild animals [4]. Raccoons are the most common carriers, followed by skunks, bats (which can significantly contribute to human cases due to their proximity to dwellings), and foxes, which act as the main reservoir for the virus in the state [4]. In November 1989, an outbreak of rabies affecting raccoons in the mid-Atlantic states spread to New Jersey [6]. Rabies in raccoons poses a significant threat to human health because, unlike other wildlife rabies reservoirs, raccoons are well adapted to coexist with people in densely populated urban and suburban areas as well as in rural areas [6]. Previous studies have used spatial epidemiologic methods to guide evidence-based rabies control efforts in the U.S. [7,8,9,10,11,12,13,14,15,16]. The New Jersey Department of Health has documented animal rabies surveillance data since 1989. To the best of our knowledge, spatial cluster analysis of animal rabies has not been performed in New Jersey. Spatial methods include descriptive disease maps [17], which visualize the spatial distribution of rabies cases, and spatial statistical tests [17] to identify spatial clusters of rabies by animal species. Knowing where rabies clusters occur allows surveillance activities to be prioritized, enabling earlier detection of outbreaks and quicker containment measures. By pinpointing areas where specific rabies-prone species congregate, wildlife vaccination programs can be strategically deployed, maximizing their impacts in high-risk zones.

Vaccinating wild animals against rabies is an important strategy for safeguarding domestic animals and the human population [18,19,20]. By implementing targeted wildlife vaccination programs, the incidence of rabies in domestic animals and human exposure to the virus can be reduced. The approach of vaccinating wildlife helps prevent the spread of rabies and decreases the economic burden associated with post-exposure prophylaxis (PEP), pet quarantine, and livestock losses [21]. Additionally, this approach contributes to broader public health goals by maintaining healthy wildlife populations and preventing potential outbreaks [21,22]. The Animal and Plant Health Inspection Service’s (APHIS) Wildlife Service (WS) program has collaborated with local, state, and federal governments; universities; and other partners in the U.S. to reduce wildlife rabies by distributing oral rabies vaccine (ORV) bait in targeted areas [23]. The U.S. National Plan for Wildlife Rabies Management 2023–2027, published in 2023 [23], serves as a 5-year framework for collaborative management of wildlife rabies in the U.S. to protect human, domestic animal, and wildlife health. The WS and its partners distribute over 8 million ORVs to wild animals like raccoons, foxes, and skunks annually through bait in the U.S. [1]. In 2022, 31,320 ORVs were distributed to raccoons in Cape May County, New Jersey [24]. The total annual cost of the animal rabies prevention system is more than USD 500 million in the U.S. [1].

The goal of our study was to identify the spatial distribution and hotspots of rabies in animals across New Jersey to gain insights into the spatial epidemiology of rabies and inform future targeted and effective animal rabies control strategies. Specifically, the objective of our study was to investigate the spatial patterns and hotspots of rabies in animals (raccoons, skunks, bats, cats, foxes, and groundhogs) at the county level in New Jersey. Identifying hotspots of rabies in animals will help allocate resources for rabies prevention and control strategies.

## 2. Materials and Methods

This retrospective ecological study investigated the incidence of rabies in animals at the county level in New Jersey, a state consisting of 21 counties [25]. The state of New Jersey is located in the mid-Atlantic and northeastern regions of the United States and is home to 9 million people [26]. New Jersey is the most densely populated state in the United States [27]. As of 2023, New Jersey’s population density is around 1300 people per square mile [28].

We focused on the animal species in New Jersey with the highest reported incidence of rabies, including raccoons, skunks, bats, cats, foxes, and groundhogs [4]. We obtained data on the yearly numbers of laboratory-confirmed [29] rabies cases by animal species in each New Jersey county from January 1989 to December 2023, courtesy of the New Jersey Department of Health [30]. The rabies cases in the New Jersey counties were aggregated for each animal species for the period from 1989 to 2023; the number of rabies cases was too small to temporally disaggregate the data. The unit of analysis was the aggregated number of laboratory-confirmed rabies cases in the animal species by county in New Jersey. We obtained a shapefile of New Jersey’s counties from an open-access data source [31].

A descriptive analysis was conducted using STATA 18.0 software (Stata Corporation, College Station, TX, USA) to examine the medians and ranges of the reported rabies cases by animal species at the county level. Choropleth maps of rabies incidence by animal species were generated for the full study period (1989–2023) using ArcGIS Pro version 3.0.3 (Environmental Systems Research Institute, Inc. (ESRI), Redlands, CA, USA). The critical intervals in the maps of rabies incidence by animal species were determined using Jenk’s optimization classification scheme [32] for the full study period (1989–2023).

We utilized spatial analytic techniques to evaluate the spatial clustering patterns of rabies at both the global and local levels [33]. Global clustering was used to examine the overall patterns of rabies in a specified area without identifying exact locations, while local clustering was used to investigate small-scale patterns of rabies across the study area [33,34].

The global Moran’s index (I), implemented in GeoDa version 1.22 [35], was used to detect significant spatial autocorrelation of rabies incidence using a queen spatial weight [33,34]. The null hypothesis was that there was no spatial clustering across the study area [36], and the analysis produced overall estimates of clustering for the entire state.

Moran local indicators of spatial association (LISA) were computed in GeoDa version 1.22 to identify the locations of significant hotspots/clusters of rabies incidence in animals using queen spatial weights [37,38]. The null hypothesis for the LISA measure was that all spatial patterns across the study area were random [37]. This analysis produced an analytical output for each county in the dataset [37]. Nine hundred ninety-nine Monte Carlo replications were used to assess statistical significance. When the simulated *p*-value was less than 0.05, the null hypothesis of no cluster was rejected [39]. Evidence of local hotspots/clusters of high rabies risk was displayed in ArcGIS Pro version 3.0.3 (Environmental Systems Research Institute, Inc (ESRI), Redlands, CA, USA) [40] using local Moran significance maps that identified local clusters with significantly high risk as well as those with significantly low risk [37].

## 3. Results

### 3.1. Spatial Patterns of Rabies Incidence by Animal Species

**Figure 1:**
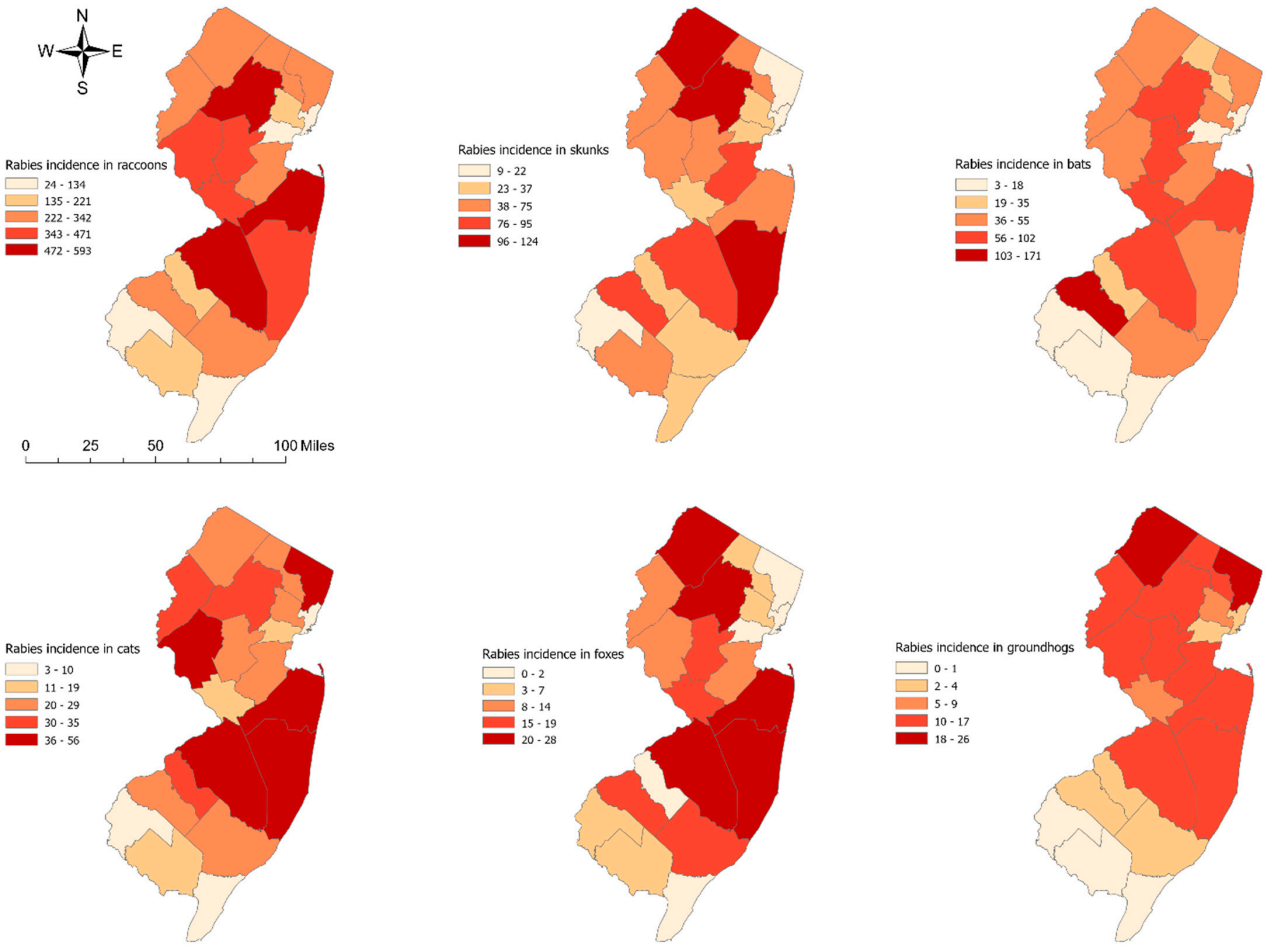
Spatial distribution of rabies incidence by animal species in New Jersey from 1989 to 2023.

A total of 9637 rabies cases were analyzed, including raccoons (n = 6308), skunks (n = 1225), bats (n = 1072), cats (n = 597), foxes (n = 225), and groundhogs (n = 210). The median rabies incidence varied by animal species across the New Jersey counties. For instance, the median rabies incidence in each county was highest in raccoons (median = 254; range: 24–593), followed by skunks (median = 59; range: 9–124), bats (median = 45; range: 3–17), cats (median = 26, range: 3–56), foxes (median = 10; range: 0–28), and groundhogs (median = 12; range: 0–26). Rabies incidence also varied geographically. In raccoons, for example, higher numbers of rabies cases tended to occur in the northern and eastern regions of New Jersey, while the lowest number of rabies cases was observed in the south (Figure 1).

### 3.2. Geographic Clusters of Rabies by Animal Species

#### 3.2.1. Global Evidence of Clustering

The global Moran’s *I* results indicate there is evidence of significant positive spatial autocorrelation of rabies cases in raccoons (Moran’s *I* = 0.32; *p* = 0.012), foxes (Moran’s *I* = 0.29; *p* = 0.011), and groundhogs (Moran’s *I* = 0.37; *p* = 0.005) (Table 1).

#### 3.2.2. Local Clusters

**Figure 2:**
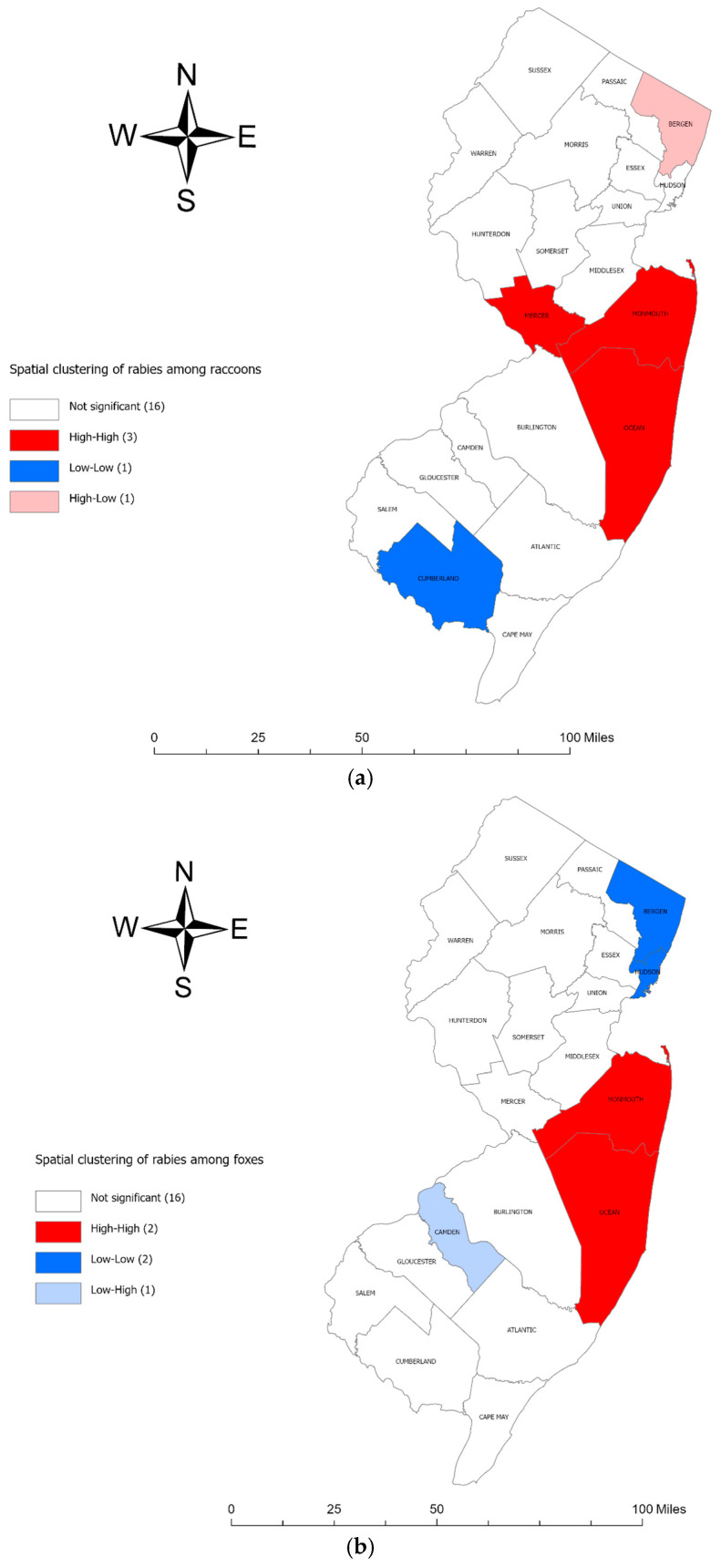

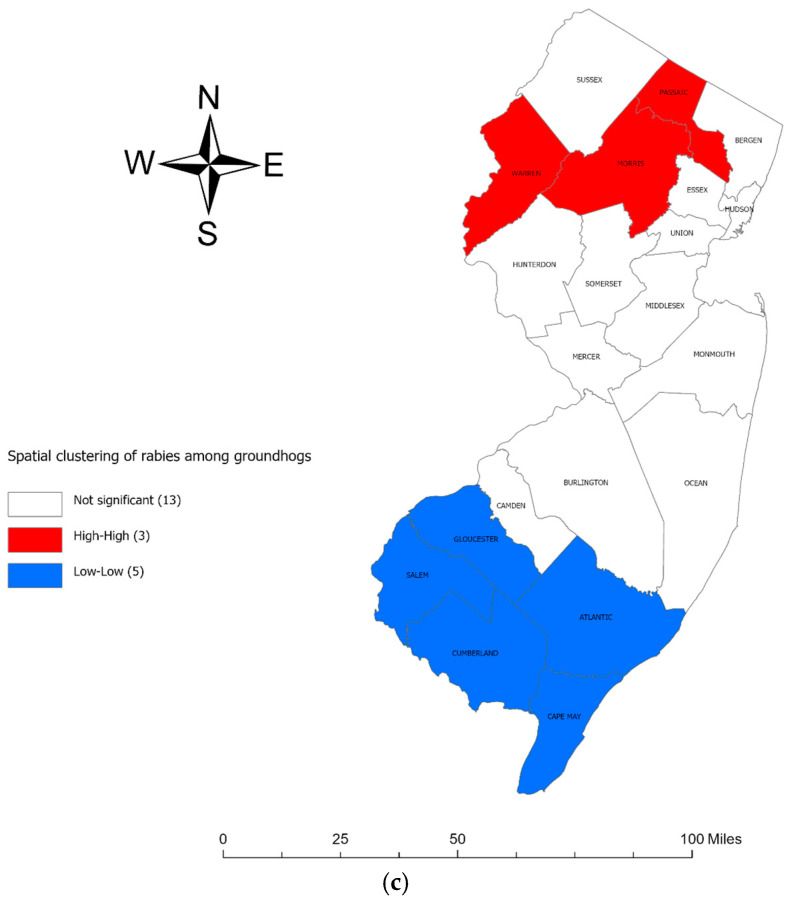
(**a**) Local Moran’s I cluster map for rabies in raccoons. Counties labeled as high–high have high incidences of rabies and are surrounded by other counties with high incidences of rabies in animals. Likewise, counties marked as low–low have low incidences of rabies and are surrounded by other counties with low incidences of rabies. A significant high-risk cluster is shown in red, while a significant low-risk cluster is shown in blue. Areas with non-significant LISA values are blank (no color shading); (**b**) Local Moran’s I cluster map for rabies in foxes; (**c**) Local Moran’s I cluster map for rabies in groundhogs.

The results of the Moran local indicators of spatial association (LISA) indicate there was a significant high-risk cluster of rabies in raccoons (Figure 2a) and foxes (Figure 2b) in counties in southeastern New Jersey. In addition, we identified a significant high-risk cluster of rabies in groundhogs (Figure 2c) in counties in northern New Jersey. Low-risk clusters were identified in southern New Jersey for raccoons and groundhogs.

## 4. Discussion

This study applied spatial methods to identify high-risk spatial clusters of rabies in animals at the county level in New Jersey. The results indicated significant global clustering of rabies in raccoons, foxes, and groundhogs across the study area. In addition, there was local spatial clustering of rabies in raccoons and foxes in southeastern counties and in groundhogs in northern counties in New Jersey.

Significant positive global spatial autocorrelation was found, indicating that rabies incidence was not randomly distributed within the study boundaries and that high and low values were more proximal to other high and low values, respectively [37].

Local spatial clustering of rabies was also detected in raccoons, foxes, and groundhogs using LISA [38]. The LISA results suggest that the southeastern counties (Ocean, Monmouth, and Mercer) in New Jersey experienced a significant high-risk spatial cluster of rabies in raccoons and foxes. The identification of raccoon rabies hotspots in Ocean, Monmouth, and Mercer Counties in southeastern New Jersey can be explained by a combination of geographic, environmental, and ecological factors. These counties have a mix of urban and suburban areas that can create ideal habitats for raccoons and foxes. A study reported that geographic and human factors such as a higher proportion of lower-intensity residential areas (those with lower concentrations of housing units), a lack of rivers/lakes, and major roads could explain the raccoon rabies clusters in Albany, New York [11,41,42]. The presence of rivers and lakes can act as a natural barrier that slows or prevents the spread of rabies among raccoon populations. On the other hand, the absence of rivers and lakes allows rabid raccoons to move more freely, which can lead to more widespread and rapid dissemination of the rabies virus. Another ecological study reported that raccoon rabies rates were explained by the higher percentages of farmland and metropolitan areas in the counties in three southeastern states (North Carolina, Virginia, and West Virginia) [8]. Jones et al. reported three characteristics associated with large raccoon rabies epizootics in Maryland, Pennsylvania, and Virginia: (1) a high percentage of agricultural land use; (2) high water coverage in combination with low human population density; and (3) low water coverage in combination with high human population density [9]. These findings were derived from a stratified analysis that revealed that the impact of the water coverage percentage on the likelihood of a county experiencing a large rabies outbreak was modified by the human population density [9]. The percentage of forest cover was associated with a raccoon rabies epidemic in Connecticut [43]. Future studies could investigate the factors related to high-risk clusters of rabies among raccoons in southeastern New Jersey.

Likewise, northern New Jersey counties (Passaic, Morris, and Warren) exhibited a significant high-risk spatial cluster of rabies in groundhogs during the study period. These counties, characterized by a mix of urban, suburban, and rural environments, provide suitable habitats for the groundhog population. Previous research indicated that northeastern and mid-Atlantic states have reported the most rabies cases in groundhogs [44], and rabid groundhogs were clustered in the U.S. counties where raccoon rabies was enzootic [45,46]. Our findings revealed a distinct spatial pattern, with rabid groundhogs clustered in northern New Jersey (Figure 2c) and rabid raccoons clustered in southeastern New Jersey (Figure 2a).

Low-risk clusters of rabies were identified among raccoons in Cumberland County (Figure 2a) and among groundhogs in Cape May, Atlantic, Cumberland, Salem, and Gloucester Counties (Figure 2c) in southern New Jersey. These counties share coastal features, including shores, beaches, and waterways, which could act as natural barriers, limiting the movement of rabid animals and reducing the spread of the disease [41]. A previous study found that crossing rivers slowed the spread of rabies by a factor of seven [47]. Another study in New York State found that each one-meter increase in land elevation and one percent increase in wetland area were linked to reduced risk of raccoon rabies [41]. However, the relationship between waterbody features and rabies incidence in raccoons and groundhogs is complex and likely influenced by other ecological and environmental factors. Another possible explanation for the low-risk rabies cluster is the impact of rabies vaccination programs on both domestic and wild animals. An oral rabies vaccination efficacy study was conducted among raccoons in Atlantic, Cumberland, and Cape May Counties, New Jersey, in April 1993 [48]. Xiaoyue Ma et al. reported a significant decrease in raccoon rabies cases following the implementation of an oral rabies vaccination program in West Virginia’s enzootic raccoon rabies areas [49]. Future studies could investigate the factors related to low-risk clusters of rabies among raccoons and groundhogs in southern New Jersey.

To our knowledge, this is the first study to investigate the spatial patterns and clusters of rabies in animals at the county level in New Jersey using spatial epidemiologic methods. The identified spatial clusters will be helpful for guiding health intervention planning and policy. Approximately 55,000 people are also administered PEP annually, resulting in over USD 200 million in healthcare costs in the U.S. [21]. Economic analyses by the WS indicate that preventing the spread of raccoon rabies in the western U.S. alone could reduce PEP costs by as much as USD 50 million annually [21]. The CDC noted that maintaining animal rabies prevention systems in the U.S. costs over USD 500 million annually [1]. Although the annual number of human rabies cases in the U.S. is relatively low (only 1–3 cases occur each year), the primary objective of targeted rabies vaccination in wild animals is to protect domestic animals and humans and minimize the economic burden associated with PEP, pet quarantine, and livestock losses [21]. Our study identified spatial clusters of rabies cases in wild animals that can aid in devising a targeted strategy for rabies vaccination in wild animals that optimizes resource allocation and minimizes costs. Targeted oral rabies vaccination programs for wildlife in areas with high-risk spatial clusters can be cost-efficient, with cost–benefit ratios greater than 1.0 [50]. However, this study is not without limitations. We were unable to analyze potential predictors associated with the areas with the highest risk (statistically significant clusters) of rabies in New Jersey due to a lack of data. Identification of localized rabies clusters could help guide future studies targeting significant rabies clusters (hotspots) and may help refine the results to improve our understanding of the contextual and geographical factors (land use type, land elevation, major roads, rivers/lakes, low-intensity residential areas, and oral rabies vaccination exposure) [11] that are associated with rabies clusters. Additionally, spatial clustering patterns can reveal areas where multiple rabies-carrying species coexist. These areas pose a higher risk of spillover to domestic animals and humans. Identifying these zones allows for targeted public education and mitigation strategies. By understanding the spatial distribution of rabies risk, public health agencies can allocate resources (vaccines and personnel) more efficiently. This includes enhanced public awareness campaigns to educate residents in hotspot areas about rabies prevention. Additionally, control strategies like wildlife population reduction might be considered in high-risk areas, but only if necessary and in accordance with responsible wildlife management practices. We were also limited in our ability to look at smaller geographic areas and shorter periods of time due to the small number of rabies cases. Therefore, our results may be limited by the modifiable area unit problem [51]. In addition, we acknowledge that analyzing the genetic evolution of wildlife rabies virus strains would be valuable and would provide additional insights into the epidemiology of rabies in New Jersey. While we were not able to include genetic analysis in this study, we believe it will be a key area for investigation in future research. A genetic analysis of wildlife rabies virus strains could help identify the sources and variations of rabies virus strains and their transmission patterns in this region, contributing to more effective control measures across different spatial scales [52,53].

These limitations notwithstanding, this study provides valuable information about the spatial variation of rabies incidence by animal species across New Jersey during the study period. This study’s findings could direct targeted oral rabies vaccination programs [19,48,54,55] in New Jersey counties with high-risk clusters of rabies among raccoons, foxes, and groundhogs to reduce the risk of rabies exposure among domestic animals and humans. Other rabies prevention activities could benefit from using the identified clustering areas. For instance, public education about exposure to raccoon, fox, and groundhog rabies and the need to increase pet vaccination activities may be prioritized in areas where clusters were identified. These programs could help control rabies epizootics in raccoons and foxes in southeastern New Jersey and groundhogs in northern New Jersey. This, in turn, would reduce the risk of rabies exposure among domestic animals and humans [6,56].

## Figures and Tables

**Table 1:** Global Moran’s *I* values and their significance test results.

Animal Species	Global Moran’s *I* (*p*-Value)
Raccoons	0.32 (0.012)
Foxes	0.29 (0.011)
Groundhogs	0.37 (0.005)
Cats	0.17 (0.071)
Bats	−0.09 (0.375)
Skunks	0.10 (0.132)

## Data Availability

The data used in this study are publicly available and can be accessed at https://www.nj.gov/health/cd/statistics/rabies-stats/ (accessed on 13 March 2024).
